# Effects of Diisocyanate Structure and Disulfide Chain Extender on Hard Segmental Packing and Self-Healing Property of Polyurea Elastomers

**DOI:** 10.3390/polym11050838

**Published:** 2019-05-08

**Authors:** Ting Li, Tianze Zheng, Jiarui Han, Zhanli Liu, Zhao-Xia Guo, Zhuo Zhuang, Jun Xu, Bao-Hua Guo

**Affiliations:** 1 Key Laboratory of Advanced Materials (MOE), Department of Chemical Engineering, Tsinghua University, Beijing 100084, China; tingli_1991@163.com (T.L.); ztz1995@outlook.com (T.Z.); chaxiangmeimei@163.com (J.H.); guozx@mail.tsinghua.edu.cn (Z.-X.G.); 2 Applied Mechanics Laboratory, School of Aerospace Engineering, Tsinghua University, Beijing 100084, China; liuzhanli@mail.tsinghua.edu.cn (Z.L.); zhuangz@tsinghua.edu.cn (Z.Z.)

**Keywords:** diisocyanate structure, disulfide bonds, hard segmental packing, self-healing, polyureas

## Abstract

Four linear polyurea elastomers synthesized from two different diisocyanates, two different chain extenders and a common aliphatic amine-terminated polyether were used as models to investigate the effects of both diisocyanate structure and aromatic disulfide chain extender on hard segmental packing and self-healing ability. Both direct investigation on hard segments and indirect investigation on chain mobility and soft segmental dynamics were carried out to compare the levels of hard segmental packing, leading to agreed conclusions that correlated well with the self-healing abilities of the polyureas. Both diisocyanate structure and disulfide bonds had significant effects on hard segmental packing and self-healing property. Diisocyanate structure had more pronounced effect than disulfide bonds. Bulky alicyclic isophorone diisocyanate (IPDI) resulted in looser hard segmental packing than linear aliphatic hexamethylene diisocyanate (HDI), whereas a disulfide chain extender also promoted self-healing ability through loosening of hard segmental packing compared to its C-C counterpart. The polyurea synthesized from IPDI and the disulfide chain extender exhibited the best self-healing ability among the four polyureas because it had the highest chain mobility ascribed to the loosest hard segmental packing. Therefore, a combination of bulky alicyclic diisocyanate and disulfide chain extender is recommended for the design of self-healing polyurea elastomers.

## 1. Introduction

Polyurea elastomers are a class of versatile materials with outstanding mechanical, anticorrosion and anti-ageing properties. They have been widely applied in industry as coatings [1,2] for various objects such as storage tanks, ships and even military trucks to protect the objects from moisture, biological deterioration [3] and chemical origin [4]. During the service time of an object, cracks inevitably occur in the coating, leading to devastation of protected object if the cracks are not repaired in a timely way [5]. However, recoating the objects is time-consuming and requires a lot of manpower and material resources. Using polyureas with good self-healing ability is an ideal strategy in dealing with cracks [3], because it can ensure autonomous repair upon occurrence of cracks with particular external stimulus. Although the self-healing of several types of polymers such as polyurethanes, poly(urethane-urea)s, polyimides and epoxy resins has been extensively investigated [6,7,8,9,10,11], only limited work deals with self-healing of polyurea elastomers [3,12]. Liu et al. developed a high performance antifouling coating that can self-heal at room temperature using a polydimethylsiloxane (PDMS)-based polyurea [3]. Our research group reported the self-healing ability of a chemically crosslinked polyurea containing disulfide bonds [12].

To design a polyurea with good self-healing ability, one must start with the microstructure of polyurea elastomers and the mechanism of self-healing property, considering the close relationship between structure and properties. Polyurea elastomers are segmented polymers that have microphase-separated structure with hard domains embedded in flexible soft matrix [13,14], similar to polyurethanes, the only difference being the urea linkage instead of urethane linkage. According to the theory of crack healing in polymers proposed by Wool and O’Connor [15], crack healing includes five stages: (a) surface rearrangements, (b) surface approach, (c) wetting, (d) diffusion, and (e) randomization. Since all these stages are closely related to chain mobility [15], the flexibility of soft segments and the level of hard segmental packing, the two important factors affecting chain mobility, are crucial for polyurea elastomers to realize rapid self-healing. In general, the soft segments are generated by the use of a long-chain diamine, typically polyether or polyester or PDMS-based diamine with molecular weights of 1000 and 2000, to ensure flexibility [3,12]. The hard segments are generated by the other two constituents, a diisocyanate and a small molecule chain-extender diamine, [16] and thus can be tailored to a great extent by varying the molecular structure of diisocyanate and chain-extender. Due to the strong intermolecular forces in the packed hard domains that restrict the chain mobility, the hard segment composition should be of great importance to implement the rearrangement of the entire molecular structures for segmented polyureas. It is generally recognized that loosely packed hard segments facilitate self-healing by increasing chain mobility [17]. Therefore, it is of great significance to investigate the effects of diisocyanate and chain extender structure on hard segmental packing and to build up a relationship between the structure of hard segment composition and self-healing property of polyurea elastomers [12,18,19].

The effects of diisocyanate structure on the microstructure and mechanical properties of segmented polyurethanes and polyureas have been investigated by Wilkes’s group using a series of symmetric and asymmetric diisocyanates [16,20,21]. They found that increased symmetry of diisocyanates resulted in more efficient hard segmental packing and the materials with symmetric hard segments have higher modulus than their asymmetric counterparts. [16] However, no work has been reported with regard to the effects of diisocyanate structure on the self-healing performance of polyurea elastomers. Even for polyurathanes, the self-healing of which has been extensively investigated, only few researchers [17] have correlated diisocyanate structure and hard segmental packing to self-healing ability. Kim et al. [17] reported that the polyurethane with an asymmetric alicyclic diisocyanate structure has better self-healing efficiency than those with symmetric alicyclic, linear aliphatic, and aromatic diisocyanate structures. However, the difference in hard segmental packing resulted from different diisocyanate structures was not investigated although it was considered and discussed, since their focus was on selecting a polyurethane with the best self-healing ability and mechanical property to develop a durable scratch-detecting electrical sensor.

The importance of chain extender structure to self-healing ability of polyurethanes has been well recognized. Chain extender containing reversible bonds are often used. The cleavage and recombination of the reversible bonds dominate the randomization process [15], the final step of self-healing process, and thus promotes the self-healing performance [7,22,23]. There are two types of reversible bonds: non-covalent and dynamic covalent bonds [24,25,26,27,28,29,30,31,32]. The dynamic covalent bond scission and recombination usually occur under certain external stimulus [24,25,27], such as UV or heating, providing the materials with unprecedented self-healing efficiency and resistance to crack. Disulfide bond (S–S) is famous among the various types of dynamic covalent bonds [24,25,26,27,28,29,30,31,32] owing to its operational feasibility, high versatility, and its low demand for external stimulus [33]. Self-healing polyurethanes containing exchangeable disulfide bonds introduced via chain extenders have drawn widespread attention as excellent self-healing materials [12,17,18,23,34]. It has been reported that both covalently crosslinked polyurethane and polyurea containing aromatic disulfides have much better self-healing ability than their C–C counterparts [12,18,35], because of the occurrence of disulfide metathesis in the hard domains [12]. However, the relationship between the disulfide bond and hard segmental packing has often been ignored. To gain a deep insight into the mechanisms behind the high self-healing performance found with the use of a disulfide chain extender, it is worth investigating the difference in hard segmental packing caused by S–S bonds.

In this work, four polyureas differing in either diisocyanate or chain extender structure are used to investigate the effects of both diisocyanate structure and aromatic disulfide chain extender on hard segmental packing and self-healing ability. The goal is to establish a relationship between the hard segment composition and the self-healing property of polyurea elastomers from the perspective of hard segmental packing. Two typical diisocyanates, i.e., a bulky alicyclic isophorone diisocyanate (IPDI) and a linear aliphatic hexamethylene diisocyanate (HDI), and two chain extenders with and without a disulfide bond, namely bis(4-aminophenyl)disulfide (AFD) and 4,4′-diaminodibenzyl (MDA), were used as the hard segment composition, and diaminopolypropylene glycol (D2000) was used as the soft segment. Direct investigation on hard segments was carried out by means of small angle X-ray scattering (SAXS), X-ray diffraction (XRD) and Fourier transform infrared spectroscopy (FTIR). The chain mobility was investigated by rheological measurements, and soft segmental dynamics was investigated by dynamic mechanical analysis (DMA), differential scanning calorimetry (DSC) and broadband dielectric spectroscopy (BDS), to provide indirect evidences on the level of hard segmental packing. Then, the self-healing property of polyurea elastomers was correlated with the diisocyanate structure and disulfide bonds on the basis of the level of hard segmental packing.

## 2. Materials and Methods

### 2.1. Materials

Diaminopolypropylene glycol (Jeffamine^®^ D2000, *M_w_* = 2000) was purchased from Huntsman International LLC (Houston, TX, USA) and dried under vacuum at 90 °C for 5 h. Isophorone diisocyanate (IPDI, 99%) and hexamethylene diisocyanate (HDI, 99%) were purchased from Aladdin (Shanghai, China). Bis(4-aminophenyl)disulfide (AFD) and 4,4’-diaminodibenzyl (MDA) were supplied by Tokyo Chemical Industry (Tokyo, Japan). Tetrahydrofuran was provided by Beijing Chemical works (Beijing, China) and freshly distilled over sodium before use.

### 2.2. Sample Preparation

**Figure 1:**
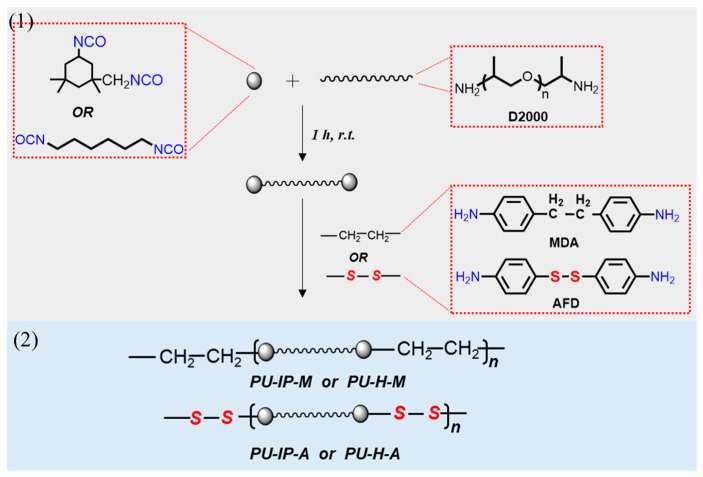
Synthetic procedures of the four different polyureas (PU-IP-A, PU-IP-M, PU-H-A and PU-H-M).

The polyureas were synthesized through the reaction between diaminopolypropylene glycol (D2000, *M_w_* = 2000) and diisocyanates (IPDI or HDI) with bis(4-aminophenyl)disulfide (AFD) or 4,4’-diaminodibenzyl (MDA) as chain extender (Figure 1). The four polyureas were denoted as PU-IP-A, PU-IP-M, PU-H-A and PU-H-M, according to the names of the corresponding diisocyanate and chain extender, where IP, H, A and M mean IPDI, HDI, AFD and MDA, respectively.

The procedure for synthesizing polyurea PU-IP-A is described as follows. To a 250 mL three-necked flask containing IPDI (1.50 g, 6.75 mmol) and tetrahydrofuran (3 mL, 50% *w*/*v*) under N_2_, was added dropwise D2000 (6.75 g, 3.37 mmol, 50% (*w*/*v*) dissolved into tetrahydrofuran). The mixture was stirred at room temperature for 1 h to obtain the isocyanate terminated prepolymer. Then, AFD (0.84 g, 3.37 mmol) was added as a chain extender. The final solution was transferred into a Teflon mold and volatilized at room temperature for 24 h. For complete drying, the mold was placed in a vacuum oven at 80 °C for 12 h to remove the residual solvent. Finally, the polyurea elastomer (PU-IP-A) sheet was obtained. An FTIR spectrophotometer was used to determine the completion of the reaction by monitoring the absorption of isocyanate around 2270 cm^−1^.

The other three polyureas were synthesized similarly. For convenience, PU-IP-A and PU-IP-M were collectively termed as IP-samples to indicate that their common feature was the use of IP as the diisocyanate, PU-H-A and PU-H-M as H-samples, PU-IP-A and PU-H-A as A-samples, and PU-IP-M and PU-H-M as M-samples.

The ^1^H NMR spectra of the samples PU-IP-A, PU-IP-M, PU-H-A and PU-H-M are shown in Figures S1–S4. FTIR spectra of all the four samples are shown in Figure S5, where the disappearance of –NCO groups indicates the complete reaction of the diisocyanates. The peaks around 1620~1720 cm^−1^ were ascribed to the C=O groups of urea bonds, confirming that the reaction products are indeed polyureas. The molecular weights of PU-IP-A, PU-IP-M and PU-H-A were measured using gel permeation chromatography (GPC) and listed in Table S1. The molecular weight of PU-H-M was absent because PU-H-M could not dissolve in the typical eluents, such as CHCl_3_ and THF, as shown in Figure S5. The similar molecular weight of the three samples reveals that the effect of molecular weight on self-healing property can be excluded.

### 2.3. Characterizations

#### 2.3.1. Fourier Transform Infrared Spectroscopy (FTIR) and Nuclear Magnetic Resonance (NMR)

All the samples for Fourier Transform Infrared Spectroscopy (FTIR) were prepared by spreading the polyurea solution onto the surface of a KBr salt tablet. The FTIR spectra were recorded on a Nicolet 6700 infrared spectrophotometer (Thermo Fisher Scientific, Waltham, MA, USA) at a resolution of 2 cm^−1^. For the temperature-dependent FTIR scans, samples were placed into a heating cell connected to a temperature controller with the temperature range from 25 to 225 °C.

The software PeakFit (PeakFit 4.12) was adopted for curve fitting procedures of carbonyl group with Gaussian function. Three typical peaks of carbonyl groups were assumed during the fitting, while the integral areas of the three peaks were obtained by peak resolving. The mean square error of all the data were smaller than 0.05.

^1^H NMR spectra were recorded on JEOL ECS-400 (Tokyo, Japan) with CDCl_3_ or CF_3_COOD as the solvents using tetramethylsilane as an internal reference.

#### 2.3.2. Gel Permeation Chromatography (GPC)

A Waters system (Waltham, MA, USA) was employed to perform GPC measurements. Tetrahydrofuran (THF) was used as eluent with a flow rate of 1 mL/min. Average molecular weights were calibrated by linear polystyrene standards.

#### 2.3.3. Dynamic Mechanical Analysis (DMA)

DMA tests were conducted on Anton Paar MCR301 (Anton Paar, Austria) with stress/strain-controlled rheometer. A torsional mode was used at a frequency of 1 Hz. The dimensions of samples are ca. 25 mm × 10 mm × 2 mm. The temperature range was from −80 to 200 °C at a heating rate of 4 °C/min. Meanwhile, the strain for the samples was 0.01% within the linear viscoelastic region.

#### 2.3.4. Stress-Relaxation Experiments

A parallel-plate mode was used with Anton Paar MCR301 (Anton Paar, Austria). Samples were cut out of the sample sheets with circular specimens with a diameter of 25 mm. Samples were compressed with a suitable normal stress to guarantee the sufficient contact between samples and plates after reaching the set temperatures. The strain was set as 5% and the strain remained unchanged during the whole process, while the stress and modulus were collected as a function of time.

#### 2.3.5. Mechanical Evaluation

Dumbbell-shaped specimens were punched out of the sample sheets according to GB/T-528. A tensile test machine (SHIMADZU AGS/X, Kyoto, Japan) was used to carry out stress−strain tests at ambient conditions at a crosshead speed of 20 mm/min.

#### 2.3.6. Small Angle X-ray Scattering (SAXS) and X-Ray Diffraction (XRD)

To characterize the microstructures of hard domains, SAXS were applied with an Xeuss system (Xenocs, Sassenage, France). The scattering experiments were carried out using a Cu point-focused source (Xenocs, Sassenage, France, *λ* = 0.154 nm) at 50 kV and 40 mA. The sample-to-detector distance was about 2500 mm. Each 2D SAXS pattern was obtained every 10 min and was averaged to get one 1D curve. Each curve should be background corrected. The interdomain spacing (*d*) was calculated with Bragg’s equation:

(1) $$d=\frac{2\pi }{q_{max}}$$

Here, *q_max_* is the peak position of the 1D curves.

XRD patterns of the four samples were collected by using a Rigaku DMAX/Rapid microdiffractometer (Rigaku, Tokyo, Japan) with a Cu point-focused source (λ = 1.54 Å).

#### 2.3.7. Thermal Analysis

Glass transition temperatures of the polyureas were characterized with a differential scanning calorimeter (DSC-60, SHIMADZU, Kyoto, Japan). The samples were heated from −90 to 230 °C at a heating rate of 20 °C/min under nitrogen atmosphere (50 mL/min). Glass transition temperature (*T_g_*) of the soft phase could be obtained from the DSC thermograms.

To evaluate the thermal stability of these materials, thermal gravimetric analysis (TGA) was conducted from 25 °C to 550 °C at a heating rate of 20 °C/min under N_2_ using DTGA-60 (SHIMADZU, Kyoto, Japan).

#### 2.3.8. Optical Analysis

To monitor crack healing, an optical microscope (Olympus BX41P, Olympus, Tokyo, Japan) with a temperature controller (Linkam T95-PE, Linkam, London, England) was used. The samples with a thickness of ca. 0.5 mm were cast on the glass slide and scratched by a razor. All samples were heated at a specified temperature for a period of time. The micrographs were captured every one minute.

#### 2.3.9. Broadband Dielectric Spectrometer (BDS)

Dielectric spectroscopy measurements were carried out to study the segmental dynamics of soft segments with a Broadband Dielectric Spectrometer (Alpha-T, Novocontrol Technologies GmbH and Co. KG, Montabaur, Germany). All the samples with a thickness of ca. 0.15 mm were gold plated and sandwiched between two gold-plated electrodes with a diameter of 10mm. Spectra were obtained in a temperature range from −70 to 10 °C with 4 °C intervals (10^−1^–10^6^ Hz).

Havriliak and Negami (HN) empirical equation [36] as below was used to analyze the dielectric spectra.

(2) $$\varepsilon^{*}\left(\omega \right){=\varepsilon }_{\infty }+\frac{\Delta \varepsilon }{{(1+{\left({i\omega \tau }_{\mathrm{NH}}\right)}^{\alpha })}^{\beta }}$$

Here, ∆*ε = ε*_0_* − ε_∞_*, is the dielectric strength; ω is the angular frequency; *ε*_0_ and *ε*_∞_ are the relaxed and unrelaxed dielectric strength. The shape parameters of the loss peak, i.e., *α* and *β*, are the overall symmetric distribution index and the asymmetric distribution index of the high frequency. The smaller is α, the wider is the width distribution of the loss peak; the larger is β, the more symmetrical is the graph curve. 0 < *α*, 0 < *αβ* <1. *τ_HN_*is the characteristic relaxation time, which is related with *τ*_max_ as follows:

(3) $$\tau_{\max}=\frac{1}{{2\pi f}_{\max}}{=\tau }_{\mathrm{HN}}{\left[\frac{\sin(\alpha \beta \pi /(2+2\beta ))}{\sin(\alpha \pi /(2+2\beta ))}\right]}^{1/\alpha }$$

Here, *f*_max_ is the frequency where ε^″^ passes through the maximum value. The *f*_max_ of the *α* processes can be correlated with a temperature-dependence behavior as expected for segmental relaxation, which follows a Vogel−Fulcher−Tammann (VFT) form as given by

(4) $$f_{\max}{=f}_{0}\exp(\frac{B}{{T-T}_{0}})$$

where *T*_0_ is the so-called Vogel temperature and the temperature coefficient (B) is related to the apparent activation energy and fragility. Here, in order to reduce fitting deviation, *f*_0_ is associated with vibration lifetimes which is fixed to 1.59 × 10^11^ Hz (*τ*_0_ = 10−12 s) for α process [37].

## 3. Results and Discussion

### 3.1. Self-Healing Ability Evaluation

**Figure 2:**
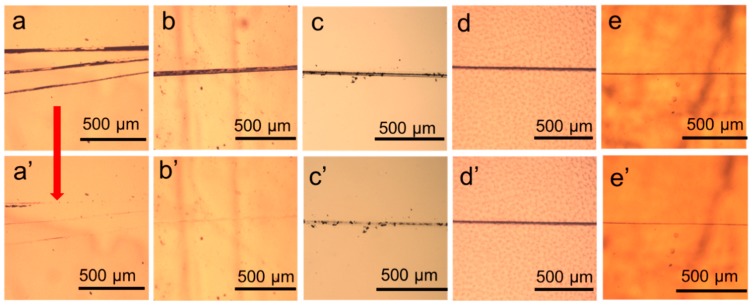
Optical images showing self-healing behavior of scratched polyureas: (**a**)–(**e**) original samples and (**a′**)–(**e′**) the heated samples. (**a**) and (**a′**) PU-IP-A before and after heating at 60 °C for 75 min, (**b**) and (**b′**) PU-IP-A before and after heating at 100 °C for 5 min, (**c**) and (**c′**) PU-IP-M before and after heating at 100 °C for 90 min, (**d**) and (**d′**) PU-H-M before and after heating at 100 °C for 90 min, (**e**) and (**e′**) PU-H-A before and after heating at 100 °C for 90 min. Scale bars are 500 μm.

The self-healing properties of the four polyureas were firstly evaluated by observing the evolution of razor scratches by means of an optical microscope. Scratched films of all the samples with a thickness of 0.5 mm were placed on the heating stage at a predetermined temperature and observed. As shown in Figure 2, the sample PU-IP-A synthesized from the bulky diisocyanate IPDI and the aromatic disulfide chain extender AFD had the best self-healing property. At 100 °C, the scars in PU-IP-A sample were no longer perceived after just 5 min (Figure 2b,b’), even at 60 °C it only needed 75 min for the scars to completely heal (Figure 2a,a’). Its non-disulfide counterpart, sample PU-IP-M, could not achieve full scratch disappearance even after 90 min at 100 °C (Figure 2c,c’), revealing the importance of disulfide moiety in promoting the self-healing ability. The scratches on the other two samples synthesized from HDI (PU-H-A and PU-H-M) had almost no change after 90 min at 100 °C (Figure 2d,d’,e,e’), indicating that the diisocyanate structure had a significant effect on the self-healing ability of polyureas. Bulky diisocyanate was more favorable to the self-healing ability of polyureas than linear aliphatic diisocyanate.

**Figure 3:**
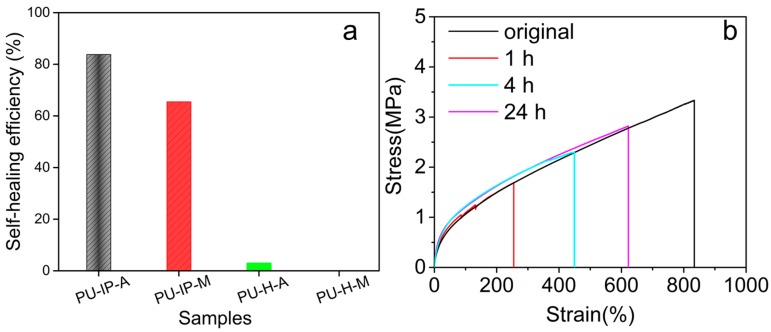
(**a**) Self-healing efficiencies of different samples after 24 h at 60 °C. (**b**) Stress–strain curves of the virgin PU-IP-A and those after healing at 60 °C for 1 h, 4 h and 24 h.

To further evaluate the self-healing efficiency, the self-healing properties were then evaluated in terms of mechanical properties by tensile tests on cut and spliced samples. Therefore, dumbbell-shaped specimens were cut into two segments with a scalpel, then the two fractured pieces were immediately stuck together and healed at 60 °C in a vacuum oven for a fixed time, and finally tensile tests were carried out to quantify the self-healing performance of all the samples. The healing temperature (60 °C) was lower than that used for the scratch tests discussed above (100 °C) because tensile testing is a more sensitive method than the scratch testing. The self-healing efficiency is defined as the ratio of the recovered ultimate toughness to the initial one, and those after 24 h of healing are summarized in Figure 3a. They decrease in the following order: PU-IP-A > PU-IP-M > PU-H-A ≈ PU-H-M, emphasizing the role of diisocyanate structure and disulfide bonds in self-healing. The sample PU-IP-A, with both bulky IPDI and disulfide moieties, exhibits the highest self-healing efficiency, and its non-disulfide counterpart (sample PU-IP-M) is the second best, being in accordance with the results of scratch recovery tests. Figure 3b shows the stress–strain curves of the PU-IP-A sample after healing for three different times (1, 4 and 24 h), indicating that the self-healing process proceeds gradually at 60 °C. Mechanical reshaping is another effective method to examine the reshuffling of polymer structures [9,38,39]. As seen from Figure S6, the two IP-samples could be remolded at 100 °C into dumbbell-shaped bars, while the other two samples (H-samples) failed, clearly indicating that the two IP-samples had better self-healing ability than the two H-samples.

To draw a general trend on the relationship between hard segment composition and self-healing ability of polyureas, the similar results from the three different types of tests mentioned above worth discussing. On the one hand, PU-H-A presented bad self-healing ability, though S-S bonds were embedded into the hard segments. On the other hand, even without disulfide bonds, PU-IP-M still had the second highest healing efficiency. Here, one is tempted to hypothesize that the diisocyanate structure should be responsible for the phenomena. As reported by Yilgor et al., hard segments with linear and symmetric HDI usually assemble into tightly packed hard domain with restricted chain segmental motion [16,20,21]. The bulky IPDI might lead to the opposite effect. Different hard segmental packing can then influence the chain mobility and subsequently the self-healing process [15]. Therefore, the choice of diisocyanates would be a promising starting point for structure design of self-healing polyureas. The conjecture shall be confirmed in the following sections. Meanwhile, with disulfide bonds and IPDI, the highest self-healing efficiency can be obtained from PU-IP-A. The exchange reaction of disulfide bonds certainly makes some contribution to the good self-healing property of PU-IP-A [12,17,23,40]. Whether the disulfide bonds affect chain mobility and hard segmental packing also deserves our further attention in the following sections to understand fully the role of disulfide bonds in self-healing.

### 3.2. Chain Mobility Analysis from a Rheological Perspective

To understand the self-healing properties in terms of chain mobility, stress-relaxation experiments were carried out from a rheological perspective. The relaxation times (*τ*) can qualitatively evaluate the self-healing properties, since they mean the recovery time of polymers after mechanical stress and can reflect the chain mobility [41].

**Figure 4:**
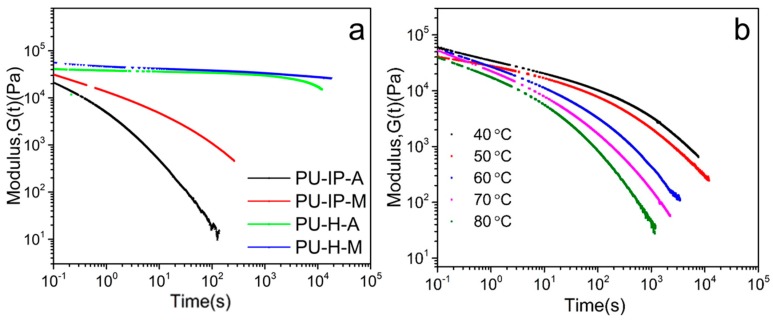
(**a**) Stress-relaxation curves of the four samples at 100 °C, (**b**) stress-relaxation curves of PU-IP-A at different temperatures.

Figure 4a shows the stress-relaxation curves of the four samples at 100 °C. The relaxation time increases in the following order: PU-IP-A < PU-IP-M < PU-H-A ≈ PU-H-M, which indicates the different chain mobility of the four samples. Here, PU-IP-A exhibits the shortest relaxation time, which correlates well with the self-healing performance shown in Section 3.1. The relaxation time of PU-IP-M is shorter than those of PU-H-A and PU-H-M, indicating that the diisocyanate structure plays a prominent role in chain mobility. Considering the importance of chain mobility in self-healing process [15], the close relationship between diisocyanate structure and self-healing property is well explained. Comparing PU-IP-A with PU-IP-M, the higher relaxation time of PU-IP-M than that of PU-IP-A indicates that the introduction of disulfide bonds can also promote chain mobility, since the only difference between PU-IP-A and PU-IP-M is the presence/absence of disulfide bonds, and the other physical effects, e.g., diffusion of polymer chains, could be excluded. In conclusion, the different chain mobility of the four samples is clearly confirmed, which is in accordance with the self-healing performance in Section 3.1.

Figure 4b presents the stress-relaxation curves of PU-IP-A at five different temperatures. The relaxation time becomes shorter when temperature increases, suggesting faster chain mobility and faster disulfide exchange reaction [40], being in agreement with the scratch recover tests where the sample recovers much faster at 100 °C than at 60 °C.

### 3.3. Hard Segmental Packing in All Samples

**Figure 5:**
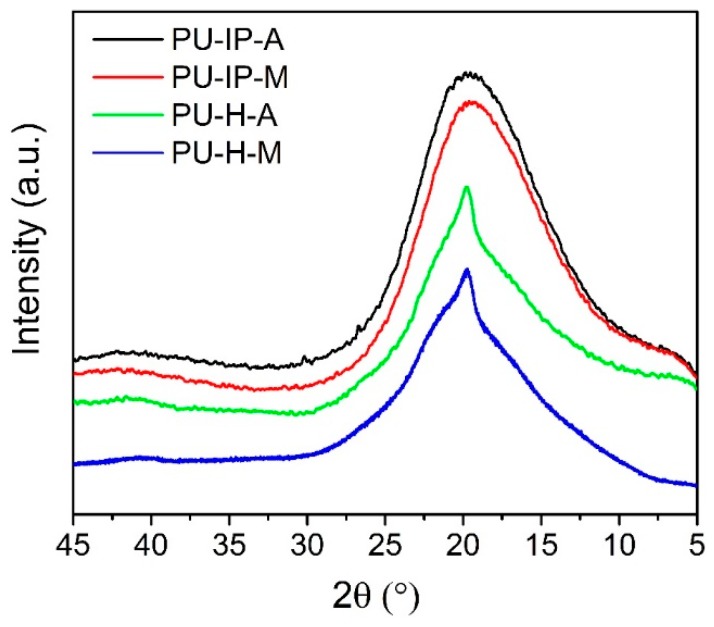
XRD patterns of the four samples.

To investigate the hard segmental structures, X-ray diffraction experiments (XRD) were firstly carried out (Figure 5). For the two IP-samples (PU-IP-A and PU-IP-M), broad scattering halos with maxima centering at ca. 2*θ* = 19° were observed (Figure 5), which suggested the amorphous form of polyureas [42]. The difference between H-samples and IP-samples was obvious. In the case of H-samples, a sharp peak on top of broad scattering halo was shown for each sample, which revealed that there might be some ordered microstructures in PU-H-A and PU-H-M. Considering the non-crystalline structure of the soft segments (D2000) with -CH_3_ as side groups (Figure S9), the sharp peaks must be derived from the tightly packed hard domains, being in line with other reports [23]. Therefore, we can speculate that the linear aliphatic structure of HDI could more easily lead to tightly packed aggregates than bulky IPDI, even in polyureas containing disulfide bonds.

**Figure 6:**
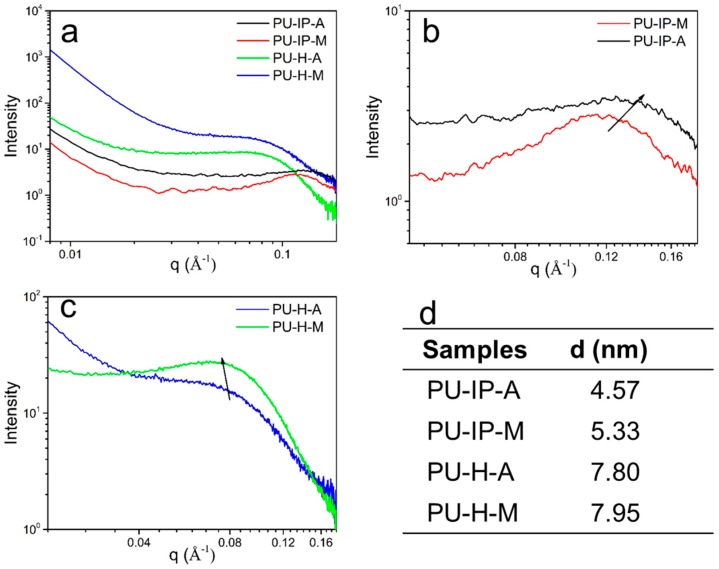
(**a**), (**b**) and (**c**) SAXS patterns (scattering intensity as a function of q) of different samples, (**d**) the mean interdomain spacings in the four samples.

To delve into the packing patterns of hard domains, the microstructures of the four samples were then investigated by SAXS experiments. Figure 6a–c displays the SAXS data of different samples with broad scattering halos, which are the typical features of randomly oriented microphase-separated structures of polyureas and polyurethanes [16,42]. Interdomain spacing (*d*) calculated by Bragg′s equation represents the distance between neighboring hard domains. As reported by Das et al. [16], polyureas with more tightly packed hard domains exhibited larger *d* than polyurethane with similar chemical segment composition but loosely packed hard domains; increased symmetry of diisocyanates in polyureas could also lead to an increase of *d* due to more tightly packed hard segments. As shown in Figure 6d, the interdomain spacing of samples increases in the following order: PU-IP-A < PU-IP-M < PU-H-A ≈ PU-H-M, implying the different levels of hard segmental packing. The larger *d* value here means the more efficient hard segmental packing [16] and stronger intermolecular forces [43,44]. Samples with the same diisocyanate show very similar *d* values, whereas samples prepared from the two different diisocyanates show very different *d* values, revealing that the effect of diisocyanate structure on hard segmental packing is more pronounced than that of disulfide bonds. Even so, the *d* values of PU-IP-M is larger than that of PU-IP-A, indicating that S-S bonds can also contribute to the loosening of hard segments. Therefore, the effects of diisocyanate structure and disulfide bonds on hard segmental packing of polyureas are both confirmed.

**Figure 7:**
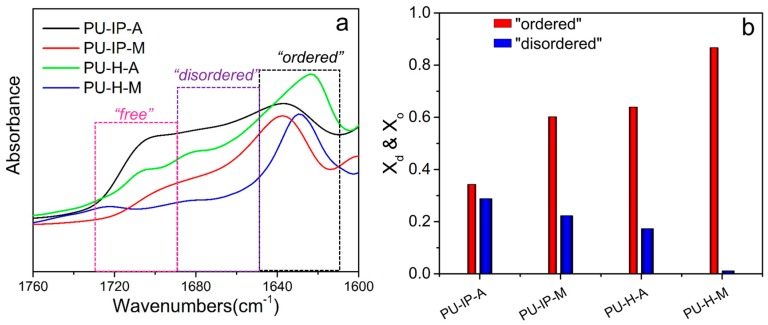
(**a**) FTIR spectra showing carbonyl groups of different samples at room temperature, (**b**) the percentages of different carbonyl groups (*X_o_* and *X_d_*) in different samples.

The packing of hard domains is also closely associated with the intermolecular forces, such as hydrogen bonds. [43,44] Observing hydrogen bonding strength was also an effective method to investigate the packing of hard domains [16,20,21,45]. The FTIR spectra showing stretching vibration of carbonyl groups (C=O) of different samples at room temperature are exhibited in Figure 7a. In general, three types of carbonyl groups are suggested in a polyurea system: “free” ones without hydrogen bonding corresponding to the highest wavenumbers, “disordered” bonds for the single ligand hydrogen bonding with reduced wavenumbers, and “ordered” ones for bidentate hydrogen bonding with the lowest wavenumbers [46]. The presence of “ordered” C=O is accompanied with regularly arranged hard domains, and denser hard domains would lead to higher content of “ordered” C=O. On the contrary, “disordered” and “free” bands originate from the loosely packed hard segments, even from the hard segments which dispersed in the soft segments [47,48]. Distinct differences in the frequency and intensity of the three types of carbonyl groups were observed for the four samples (Figure 7a). On the one hand, the hydrogen bonding strength of the two IP-samples was weaker than that of the two H-samples as suggested by the higher wavenumbers (1638 cm^−1^ vs 1633 cm^−1^), implying the role of diisocyanate structures on hard segmental packing. On the other hand, as revealed by the higher intensities of “disordered” and “free” hydrogen bonding of A-samples compared to those of the corresponding M-samples (PU-IP-A vs PU-IP-M, PU-H-A vs PU-H-M), the disulfide bonds could loosen hard segmental packing. Therefore, a proper choice of diisocyanates might be more important than using a disulfide chain extender for loosening hard segmental packing in order to have good self-healing ability.

To quantitatively compare the difference in hydrogen bonding behavior in different samples, the percentages of the three types of carbonyl groups, *X_o_*, *X_d_*, and *X_f_*, were calculated using the following equations, supposing that the absorption coefficients for carbonyl groups are 1.0. [12,45,49,50]

(5) $$X_{f}=\frac{A_{f}}{A_{f}+A_{d}+A_{o}}$$

(6) $$X_{d}=\frac{A_{d}}{A_{f}+A_{d}+A_{o}}$$

(7) $$X_{o}=\frac{A_{o}}{A_{f}+A_{d}+A_{o}}$$

Here, *A_o_*, *A_d_*, and *A_f_* refer to the peak areas of “ordered”, “disordered” and “free” carbonyl groups, respectively, while *X_o_*, *X_d_*, and *X_f_* refers to the percentages of the corresponding carbonyl bands. Here, *X_o_* can be taken as a representative of the hard segmental packing.

The values of *X_o_* and *X_d_* are given in Figure 7b. *X_o_* increases in the following order: PU-IP-A < PU-IP-M < PU-H-A < PU-H-M, whereas *X_d_* shows opposite order. The *X_o_* values of the IP-samples are indeed much lower than those of the corresponding H-samples, and the *X_o_* values of the A-samples are much lower than those of the corresponding M-samples, supporting the visual estimation discussed above, and this proves that both diisocyanate and disulfide chain extender are important factors to be considered in the design of self-healing polyureas.

**Figure 8:**
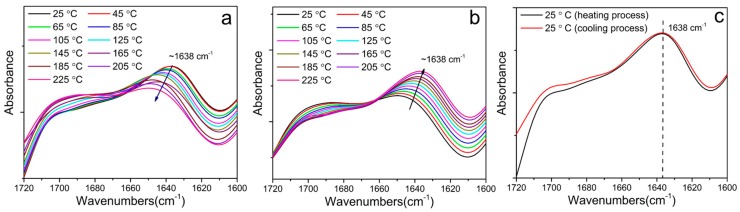
(**a**) and (**b**) Temperature-dependent FTIR spectra showing carbonyl groups of PU-IP-A during the heating and cooling process, respectively. (**c**) Comparison of carbonyl bands in FTIR spectra of PU-IP-A at 25 °C before heating and after cooling to 25 °C.

The evolution of hydrogen bonding strength during heating and cooling processes can illustrate the destruction and reconstruction of the polymer microstructure and therefore reflect the practical healing process under thermal stimulus. During the heating and cooling processes, the frequency and intensity of the peak of ordered carbonyl groups are indications of hydrogen bonding strength. Therefore, the sample PU-IP-A that had the best self-healing ability was selected as a representative to carry out the investigation by monitoring the change of “ordered” carbonyl groups as a function of temperature in order to understand the healing process at the molecular level. As shown in Figure 8a,b, red shifting of the wavenumber and a declined intensity occur with increasing temperature, whereas blue shifting of the wavenumber and an increased intensity occur with decreasing temperature, revealing that hydrogen bonding strength gradually decreases when the temperature increases and increases when the temperature decreases. Figure 8c shows the comparison of the carbonyl groups in the PU-IP-A analyzed at the initial 25 °C before heating and at the final 25 °C after the cooling process. The complete reversibility of the hydrogen bonding for PU-IP-A is observed, which reflects the complete structural recovery. On the contrary, Figure S8 shows the irreversibilities of hydrogen bonding for the other three samples (PU-IP-M, PU-H-A and PU-H-M). This indicates that the sample PU-IP-A synthesized using a combination of bulky IPDI and disulfide chain extender can self-heal upon heating and cooling in a practical sense, further confirming that the disulfide bonds also make important contribution to the self-healing performance of polyureas although the diisocyanate structure has more pronounced effect than the disulfide chain extender.

### 3.4. Soft Segmental Dynamics

In a typical microphase-separated structure, soft segments must be greatly influenced by the hard segments, since they are directly linked with each other. Generally, if hard domains are loosely packed, more hard segments will penetrate into soft phase, leading to the hindered soft segments, which results in an increase of *T*_g_ of the soft segments [17,51]. Therefore, *T*_g_ of the soft segments was measured by three methods (DMA, DSC and BDS) in this work to provide indirect information on the level of hard segmental packing.

**Figure 9:**
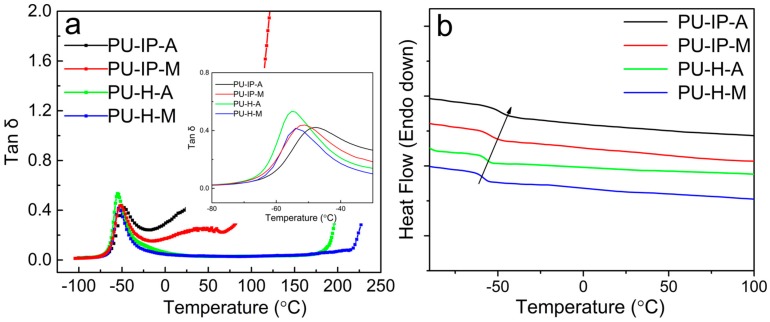
(**a**) Damping factor as a function of temperature for the four samples. (**b**) DSC curves (heat flow dependent on temperature) of the four polyureas.

The damping factor-temperature curves obtained from DMA are presented in Figure 9a, showing obvious *α* relaxations of soft segments for all the samples. The values of *T*_g_ are listed in Table 1. They are all higher than that of the soft segment composition used for polycondensation (polyether diamine D2000, −67 °C, Figure S9) because of the restricted soft segmental motion by the hard segments. The *T*_g_s of the two H-samples (−55.2 and −54.2 °C) are much lower than those of the IP-samples (−47.7 and −51.1 °C). This indicates that the soft segments of the H-samples are farther from the hard segments than those of the IP-samples, i.e., the hard segmental packing is much tighter in H-samples than in IP-samples, revealing the great influence of diisocyanate structure on the hard segmental packing. In addition, the role of disulfide bonds in loosening hard segmental packing can also be confirmed from the comparison between PU-IP-A and PU-IP-M. The four *T*_g_s in decreasing order are: PU-IP-A > PU-IP-M > PU-H-A ≈ PU-H-M, indicating that the degree of hard segmental packing is in a reverse order, being basically in agreement with the results of the aforementioned investigation on hard segments. The *T*_g_ values obtained from DSC measurements are in line with those obtained from DMA measurements within the experimental errors (Figure 9b and Table 1).

**Figure 10:**
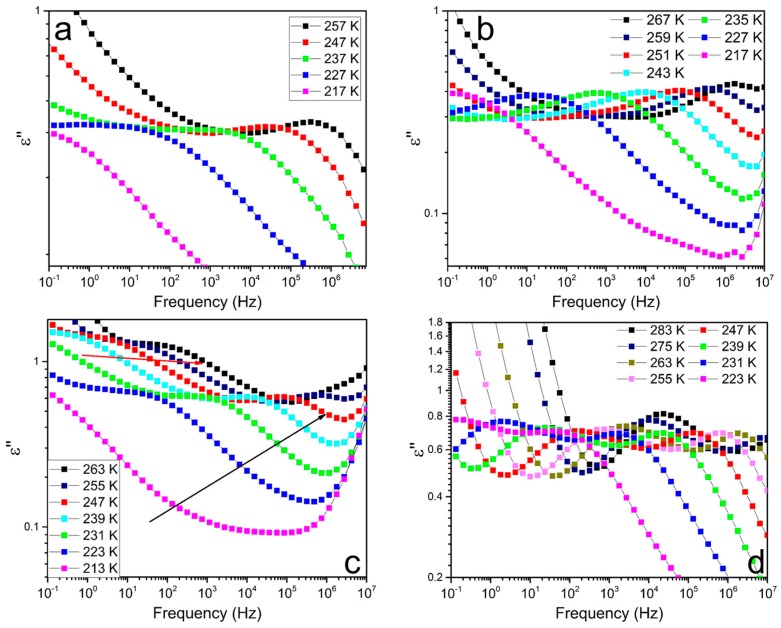
Relaxation behaviors of PU-IP-A (**a**), PU-IP-M (**b**), PU-H-A (**c**), and PU-H-M (**d**).

Broadband dielectric spectrometer (BDS) is a powerful tool for studying polymer structure and molecular motion [52,53,54]. To further investigate the soft segmental dynamics, BDS was used over a broad frequency and proper temperature range. Figure 10 depicts the frequency and temperature dependence of dielectric loss processes for the four samples. At the lowest frequencies a pronounced increase in *ε*′′ is observed for all the samples; this is due to dc conductivity and interfacial polarization at the boundaries between soft and hard domains, which have different dielectric constants and conductivities. With frequency increasing, different relaxation peaks of the four samples emerge. As shown in Figure 10a,b, the two IP-samples (PU-IP-A and PU-IP-M) show similar relaxation behaviors with only one broad peak in each sample, which might reflect the *α* relaxation (segmental motion of soft phase); whereas two peaks are observed for each H-samples (PU-H-A and PU-H-M) as shown in Figure 10c,d, one peak might be representative of the typical *α* relaxation, and the other could be designated as *α*′ relaxation.

**Figure 11:**
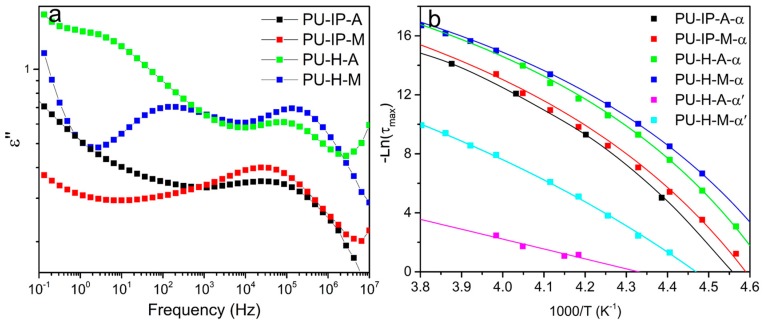
The comparison of dielectric analysis between α relaxation and α′ relaxation for the four samples. (**a**) *ε″* as a function of frequency. (**b**) Temperature dependence of *τ*_max_ for *α* relaxation and *α′*. Solid lines represent fitting curves.

To identify and ascertain *α* and *α’* relaxations, the frequency-dependent spectra of the four samples at 247 K are selected as representatives (Figure 11a). Comparing the four samples, the peaks with the similar positions in the higher frequency region (10^4^~10^6^ Hz) for all the samples might refer to α relaxation; the new peaks in the lower frequency region (10^1^~10^2^ Hz) only for PU-H-A and PU-H-M might correspond to the *α′* relaxation. *τ*_max_ of all the peaks were then obtained from the fitting process with Havriliak–Negami equation. The temperature-dependent *τ*_max_ could be fitted with VFT equation, as shown in Figure 11b. To further confirm the nature of all the peaks, we obtained *T_g_* for all the peaks via extrapolating the VFT fit to *τ* = 100 s, [37] and the values are presented in Table 1. The *T_g_* values for *α* relaxation of all the samples match well with those obtained from other testing methods (DMA and DSC) within the allowable range of experimental errors, further confirming the facticity of *α* relaxation. Meanwhile, the *T_g_* values of *α*’ relaxation for PU-H-A and PU-H-M are higher than those of *α* relaxation, indicating the slower soft segmental dynamics (*i.e.,* more restricted soft segments).

The difference in peak numbers between IP- and H-samples might result from the differently packed hard domains. Generally, the microphase separation of polyureas is incomplete, so there are two types of soft segments: the ones directly jointed with the hard domains (i.e., the interfacial region between a soft domain and a hard domain) and the others far from the hard domains and dispersed in the soft phase, indicating the different degrees to be restricted for soft segments by different hard segmental packing [51,55]. If the hard domains tightly pack, the soft segments separate well from the hard domains and the distinction between the two types of soft segments is relatively big and might be distinguished, just like the case of H-samples. However, in case of loosely packed hard domains, it is hard to distinguish the two types of soft segments because of the great microphase-mixing between the hard and soft segments. Therefore, we can explain why there is only one peak in samples PU-IP-A and PU-IP-M and two peaks in samples PU-H-A and PU-H-M. BDS is not sensitive enough to distinguish *α*′ relaxation and *α* relaxation of IP samples because the two peaks overlap, as seen by the wider transition region for PU-IP-A and PU-IP-M than for PU-H-A and PU-H-M in Figure 11. The effect of diisocyanate structure on hard segmental packing is confirmed again.

After identifying the relaxations, the main task for us is to distinguish the differences of soft segmental dynamics among the four samples, as presented in Figure 11a,b. In terms of *α* relaxation in Figure 11a, *f*_max_ (the frequency where *ε″* passes through the maximum value) increases in the following order: PU-IP-A < PU-IP-M < PU-H-A < PU-H-M. Since the lower *f*_max_ indicates the more restricted segments, the *f*_max_ order is essentially consistent with the degree of soft segments to be hindered. The same order of soft segmental dynamics can also be clearly demonstrated from the VFT fitting in Figure 11b. The order is exactly consistent with that of the packing degree of hard segments. From the order of soft segmental dynamics, the roles of diisocyanate structure and disulfide bonds are confirmed again. With bulky IPDI, the IP-samples exhibit slower soft segmental dynamics than H-samples, reflecting that their level of hard segmental packing is lower than that of H-samples. The introduction of disulfide bonds can also loosen the hard domains and restrict the soft segmental dynamics, as revealed by comparing the A-samples with M-samples. In addition, comparing *α*′ relaxations of PU-H-A and PU-H-M in Figure 11b, the *α*′ process of PU-H-A is much slower than that of PU-H-M, further confirming the role of disulfide bonds in loosening the hard segmental packing. In conclusion, we can deduce that both *α* relaxation and *α*′ relaxation elucidate soft segmental dynamics and reflect hard segmental packing, and the results are in accordance with the aforementioned data (Section 3.3).

## 4. Conclusions

Direct investigation on hard segments and indirect reflection by chain mobility and soft segmental dynamics lead to agreed conclusions about the effects of diisocyanate structure and aromatic disulfide chain extender on hard segmental packing, which correlates well with the self-healing ability of different polyureas. Both disulfide bond and diisocyanate structure are very important factors affecting hard segmental packing. The diisocyanate structure is confirmed to have a greater influence than disulfide bonds. A combination of bulky steric diisocyanate structure and disulfide bonds favors loose packing of hard segments and endows polyureas with good self-healing ability. Among the four polyureas investigated in this work, the one prepared from bulky diisocyanate and disulfide chain extender has the loosest hard segmental packing and the best self-healing ability even under mild conditions (60 °C). This work can provide an important guidance for the design of polyureas with excellent self-healing ability at the molecular level.

## Figures and Tables

**Table 1:** Comparison of *T_g_* values obtained from DMA, DSC and BDS.

Samples	PU-IP-A	PU-IP-M	PU-H-A	PU-H-M
*T*_g,DMA_ (°C)	−47.7	−51.1	−55.2	−54.2
*T*_g,DSC_ (°C)	−47.3	−52.9	−56.2	−58.2
*T*_g,DRS_ (°C)	−54.6	−58.6	−60.2	−60.3
*T*_g,DRS,α’_ (°C)	--	--	−52.3	−54.0

## Supplementary Materials

The following are available online at https://www.mdpi.com/2073-4360/11/5/838/s1. Figure S1: ^1^H NMR spectrum (in CDCl_3_) of PU-IP-A, Figure S2: ^1^H NMR spectrum (in CDCl_3_) of PU-IP-M, Figure S3: ^1^H NMR spectrum (in CF_3_COOD) of PU-H-A, Figure S4: ^1^H NMR spectrum (in CF_3_COOD) of PU-H-M, Table S1: Molecular weights of samples, Figure S5; Different solubilities of four polyureas in DMF after 24 h, Figure S6: FTIR spectra of PU-IP-A, PU-IP-M, PU-H-M, and PU-H-A, Figure S7: Mechanical reshaping of different samples, Figure S8: TGA profiles for the four samples, Figure S9: FTIR spectra showing carbonyl groups of PU-IP-M, PU-H-A and PU-H-M at 25 °C before heating and after cooling to 25 °C, Figure S10: DSC curve of the soft constituent D2000.

## References

[1] Qian X., Song L., Tai Q., Hu Y., Yuen R.K. (2013). Graphite oxide/polyurea and graphene/polyurea nanocomposites: A comparative investigation on properties reinforcements and mechanism. Compos. Sci. Technol..

[2] Casalini R., Bogoslovov R., Qadri S., Roland C. (2012). Nanofiller reinforcement of elastomeric polyurea. Polymer.

[3] Liu C., Xie Q., Ma C., Zhang G. (2016). Fouling Release Property of Polydimethylsiloxane Based Polyurea with Improved Adhesion to Substrate. Ind. Eng. Chem. Res..

[4] Chattopadhyay D.K., Raju K.V.S.N. (2007). Structural engineering of polyurethane coatings for high performance applications. Prog. Polym. Sci..

[5] Yang W.J., Tao X., Zhao T., Weng L., Kang E.T., Wang L.H. (2015). Antifouling and Antibacterial Hydrogel Coatings with Self-healing Properties Based on Dynamic Disulfide Exchange Reaction. Polym. Chem..

[6] Liu C., Ma C., Xie Q., Zhang G. (2017). Self-repairing silicone coating for marine anti-biofouling. J. Mater. Chem. A.

[7] Chen X., Dam M.A., Ono K., Mal A., Shen H., Nutt S.R., Sheran K., Wudl F. (2002). A thermally re-mendable cross-linked polymeric material. Science.

[8] Lu Y.-X., Tournilhac F., Leibler L., Guan Z. (2012). Making insoluble polymer networks malleable via olefin metathesis. J. Am. Chem. Soc..

[9] Lei Z.Q., Xie P., Rong M.Z., Zhang M.Q. (2015). Catalyst-free dynamic exchange of aromatic Schiff base bonds and its application to self-healing and remolding of crosslinked polymers. J. Mater. Chem. A.

[10] Zhang Z.P., Rong M.Z., Zhang M.Q., Yuan C.E. (2013). Alkoxyamine with reduced homolysis temperature and its application in repeated autonomous self-healing of stiff polymers. Polym. Chem..

[11] De Luzuriaga A.R., Martin R., Markaide N., Rekondo A., Cabañero G., Rodríguez J., Odriozola I. (2016). Epoxy resin with exchangeable disulfide crosslinks to obtain reprocessable, repairable and recyclable fiber-reinforced thermoset composites. Mater. Horiz..

[12] Li T., Xie Z., Xu J., Weng Y., Guo B.-H. (2018). Design of a self-healing cross-linked polyurea with dynamic cross-links based on disulfide bonds and hydrogen bonding. Eur. Polym. J..

[13] Rinaldi R., Boyce M., Weigand S., Londono D., Guise M. (2011). Microstructure evolution during tensile loading histories of a polyurea. J. Polym. Sci. Part B Polym. Phys..

[14] Castagna A.M., Pangon A., Dillon G.P., Runt J. (2013). Effect of Thermal History on the Microstructure of a Poly (tetramethylene oxide)-Based Polyurea. Macromolecules.

[15] Wool R.P., O’Connor K.M. (1981). A theory crack healing in polymers. J. Appl. Phys..

[16] Das S., Cox D.F., Wilkes G.L., Klinedinst D.B., Yilgor I., Yilgor E., Beyer F. (2007). Effect of Symmetry and Hydrogen bond Strength of Hard Segments on the Structure Property Relationships of Segmented, Nonchain Extended Polyurethanes and Polyureas. J. Macromol. Sci. Part B.

[17] Kim S.M., Jeon H., Shin S.H., Park S.A., Jegal J., Hwang S.Y., Oh D.X., Park J. (2018). Superior Toughness and Fast Self-Healing at Room Temperature Engineered by Transparent Elastomers. Adv. Mater..

[18] Martin R., Rekondo A., de Luzuriaga A.R., Cabañero G., Grande H.J., Odriozola I. (2014). The processability of a poly (urea-urethane) elastomer reversibly crosslinked with aromatic disulfide bridges. J. Mater. Chem. A.

[19] Zhang L., Chen L., Rowan S.J. (2017). Trapping dynamic disulfide bonds in the hard segments of thermoplastic polyurethane elastomers. Macromol. Chem. Phys..

[20] Sami S., Yildirim E., Yurtsever M., Yurtsever E., Yilgor E., Yilgor I., Wilkes G.L. (2014). Understanding the influence of hydrogen bonding and diisocyanate symmetry on the morphology and properties of segmented polyurethanes and polyureas: Computational and experimental study. Polymer.

[21] Sheth J.P., Klinedinst D.B., Wilkes G.L., Yilgor I., Yilgor E. (2005). Role of chain symmetry and hydrogen bonding in segmented copolymers with monodisperse hard segments. Polymer.

[22] Chen X., Wudl F., Mal A.K., Shen H., Nutt S.R. (2003). New thermally remendable highly cross-linked polymeric materials. Macromolecules.

[23] Xu W.M., Rong M.Z., Zhang M.Q. (2016). Sunlight driven self-healing, reshaping and recycling of robust, transparent and yellowing-resistant polymer. J. Mater. Chem. A.

[24] Burattini S., Colquhoun H.M., Fox J.D., Friedmann D., Greenland B.W., Harris P.J., Hayes W., Mackay M.E., Rowan S.J. (2009). A self-repairing, supramolecular polymer system: Healability as a consequence of donor-acceptor pi-pi stacking interactions. Chem. Commun..

[25] Ono T., Fujii S., Nobori T., Lehn J.M. (2007). Soft-to-hard transformation of the mechanical properties of dynamic covalent polymers through component incorporation. Chem. Commun..

[26] Hentschel J., Kushner A.M., Ziller J., Guan Z. (2012). Self-healing supramolecular block copolymers. Angew. Chem. Int. Ed..

[27] Varley R.J., Sybrand V.D.Z. (2010). Autonomous damage initiated healing in a thermo-responsive ionomer. Polym. Int..

[28] Zhang Y., Dai Z., Han J., Li T., Xu J., Guo B.H. (2017). Interplay between crystallization and Diels-Alder reaction in biobased multiblock copolyesters possessing dynamic covalent bond. Polym. Chem..

[29] Zhang D.D., Ruan Y.B., Zhang B.Q., Qiao X., Deng G., Chen Y., Liu C.Y. (2017). A self-healing PDMS elastomer based on acylhydrazone groups and the role of hydrogen bonds. Polymer.

[30] Zheng N., Fang Z., Zou W., Zhao Q., Xie T. (2016). Thermoset Shape-Memory Polyurethane with Intrinsic Plasticity Enabled by Transcarbamoylation. Angew. Chem. Int. Ed..

[31] Yuan C.E., Rong M.Z., Zhang M.Q., Zhang Z.P., Yuan Y.C. (2011). Self-Healing of Polymers via Synchronous Covalent Bond Fission/Radical Recombination. Chem. Mater..

[32] Imato K., Kanehara T., Nojima S., Ohishi T., Higaki Y., Takahara A., Otsuka H. (2016). Repeatable mechanochemical activation of dynamic covalent bonds in thermoplastic elastomers. Chem. Commun..

[33] Ohishi T., Iki Y., Imato K., Higaki Y., Takahara A., Otsuka H. (2013). Insertion Metathesis Depolymerization of Aromatic Disulfide-containing Dynamic Covalent Polymers under Weak Intensity Photoirradiation. Chem. Lett..

[34] Xu Y., Chen D. (2016). A novel self-healing polyurethane based on disulfide bonds. Macromol. Chem. Phys..

[35] Grande A.M., Martin R., Odriozola I., Zwaag S.V.D., Garcia S.J. (2017). Effect of the polymer structure on the viscoelastic and interfacial healing behaviour of poly (urea-urethane) networks containing aromatic disulphides. Eur. Polym. J..

[36] Havriliak S., Negami S. (1967). A complex plane representation of dielectric and mechanical relaxation processes in some polymers. Polymer.

[37] Yu W., Du M., Zhang D., Lin Y., Zheng Q. (2013). Influence of dangling chains on molecular dynamics of polyurethanes. Macromolecules.

[38] Lei Z.Q., Xiang H.P., Yuan Y.J., Rong M.Z., Zhang M.Q. (2014). Room-Temperature Self-Healable and Remoldable Cross-linked Polymer Based on the Dynamic Exchange of Disulfide Bonds. Chem. Mater..

[39] Montarnal D., Capelot M., Tournilhac F., Leibler L. (2011). Silica-like malleable materials from permanent organic networks. Science.

[40] Lai Y., Kuang X., Zhu P., Huang M., Dong X., Wang D. (2018). Colorless, Transparent, Robust, and Fast Scratch-Self-Healing Elastomers via a Phase-Locked Dynamic Bonds Design. Adv. Mater..

[41] Kwon Y.J., Hong S.M., Koo C.M. (2010). Viscoelastic properties of decrosslinked irradiation-crosslinked polyethylenes in supercritical methanol. J. Polym. Sci. Part B Polym. Phys..

[42] Wang C.B., Cooper S.L. (1983). Morphology and properties of segmented polyether polyurethaneureas. Macromolecules.

[43] Versteegen R.M., Kleppinger R., Sijbesma R.P., Meijer E.W. (2005). Properties and Morphology of Segmented Copoly(ether urea)s with Uniform Hard Segments. Macromolecules.

[44] Kautz H., Beek D.J.M.V., Sijbesma R.P., Meijer E.W. (2006). Cooperative End-to-End and Lateral Hydrogen-Bonding Motifs in Supramolecular Thermoplastic Elastomers. Macromolecules.

[45] Li T., Zhang C., Xie Z., Xu J., Guo B.-H. (2018). A multi-scale investigation on effects of hydrogen bonding on micro-structure and macro-properties in a polyurea. Polymer.

[46] And J.M., Painter P. (2007). A Comparison of Hydrogen Bonding and Order in a Polyurethane and Poly(urethane−urea) and Their Blends with Poly(ethylene glycol). Macromolecules.

[47] Coleman M.M., Lee K.H., Skrovanek D.J., Painter P.C. (1986). Hydrogen bonding in polymers. 4. Infrared temperature studies of a simple polyurethane. Macromolecules.

[48] Han S.L., Ying K.W., Hsu S.L. (1988). Spectroscopic analysis of phase separation behavior of model polyurethanes. Macromolecules.

[49] Teo L.S., Chuhyung Chen A., Kuo J.F. (1997). Fourier Transform Infrared Spectroscopy Study on Effects of Temperature on Hydrogen Bonding in Amine-Containing Polyurethanes and Poly(urethane−urea)s. Macromolecules.

[50] Srichatrapimuk V., Cooper S. (2012). Infrared thermal analysis of polyurethane block polymers. J. Macromol. Sci. Part B.

[51] Fragiadakis D., Gamache R., Bogoslovov R., Roland C. (2010). Segmental dynamics of polyurea: Effect of stoichiometry. Polymer.

[52] Raftopoulos K., Pandis C., Apekis L., Pissis P., Janowski B., Pielichowski K., Jaczewska J. (2010). Polyurethane–POSS hybrids: Molecular dynamics studies. Polymer.

[53] Castagna A.M., Fragiadakis D., Lee H., Choi T., Runt J. (2011). The role of hard segment content on the molecular dynamics of poly (tetramethylene oxide)-based polyurethane copolymers. Macromolecules.

[54] Luo M.C., Zhang X.K., Zeng J., Gao X.X., Huang G.S. (2016). Enhanced relaxation behavior below glass transition temperature in diene elastomer with heterogeneous physical network. Polymer.

[55] Castagna A.M., Wang W., Winey K.I., Runt J. (2011). Structure and dynamics of zinc-neutralized sulfonated polystyrene ionomers. Macromolecules.

