# Supplementary material for: Effects of Diisocyanate Structure and Disulfide Chain Extender on Hard Segmental Packing and Self-Healing Property of Polyurea Elastomers
Source: Polymers (Basel). 2019 May 8;11(5):838. doi: 10.3390/polym11050838 (PMC6572514; doi:10.3390/polym11050838)
Supplement: Supplementary file 1 [file polymers-11-00838-s001.pdf]

Effects of Diisocyanate Structure and Disulfide Chain Extender on Hard Segmental Packing and Self-healing Property of Polyurea Elastomers

Ting Li [a], Tianze Zheng [a], Jiarui Han [a], Zhanli Liu [b], Zhao-Xia Guo [a], Zhuo Zhuang [b], Jun Xu [a*], and Bao-Hua Guo [a*]

a. Key Laboratory of Advanced Materials (MOE), Department of Chemical Engineering, Tsinghua University, Beijing 100084, P. R. China

b. Applied Mechanics Laboratory, School of Aerospace Engineering, Tsinghua University, Beijing 100084, China

Corresponding authors: Bao-Hua Guo and Jun Xu

E-mails: bhguo@mail.tsinghua.edu.cn, jun-xu@mail.tsinghua.edu.cn

Tel: +86-10-62784550

Supplementary Information

Figure S1. ^1H NMR spectrum (in $CDCl_3$) of PU-IP-A.

Figure S2. ^1H NMR spectrum (in $CDCl_3$) of PU-IP-M.

Figure S3. ^1H NMR spectrum (in CF_3COOD) of PU-H-A.

Figure S4. ^1H NMR spectrum (in CF_3COOD) of PU-H-M.

Table S1. Molecular weights of samples.

Figure S5. Different solubilities of four polyureas in DMF after 24 h.

Figure S6. FTIR spectra of PU-IP-A, PU-IP-M, PU-H-M, and PU-H-A.

Figure S7. Mechanical reshaping of different samples.

Figure S8. TGA profiles for the four samples.

Figure S9. FTIR spectra showing carbonyl groups of PU-IP-M, PU-H-A and PU-H-M at 25 °C before heating and after cooling to 25 °C.

Figure S10. DSC curve of the soft constituent D2000.

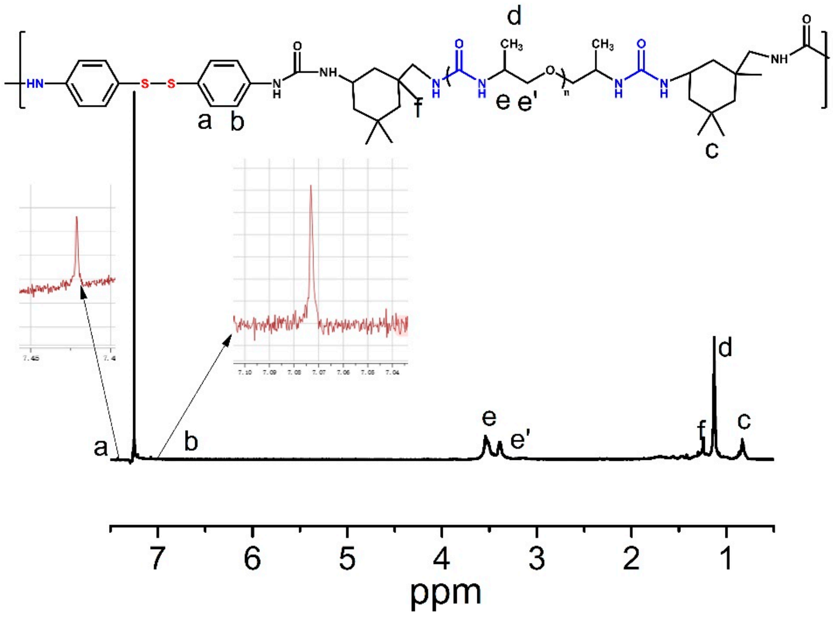

Figure S1. ^1H NMR spectrum (in $CDCl_3$) of PU-IP-A.

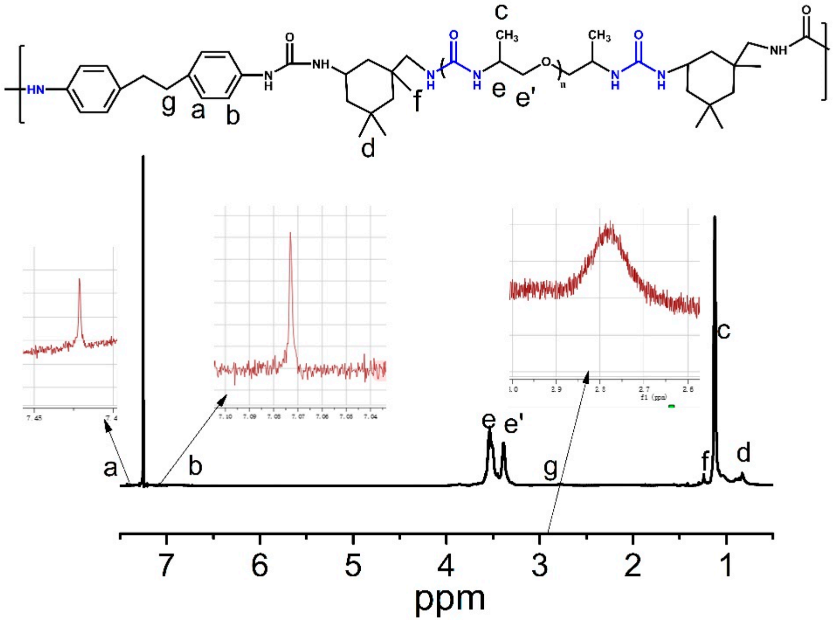

Figure S2. ^1H NMR spectrum (in $CDCl_3$) of PU-IP-M.

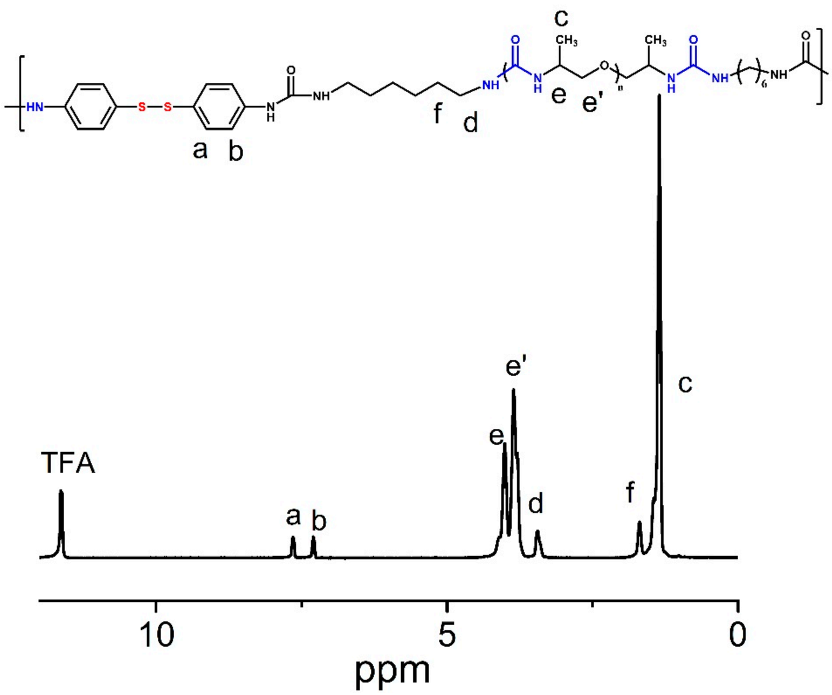

Figure S3. ^{1}H NMR spectrum (in CF3COOD) of PU-H-A.

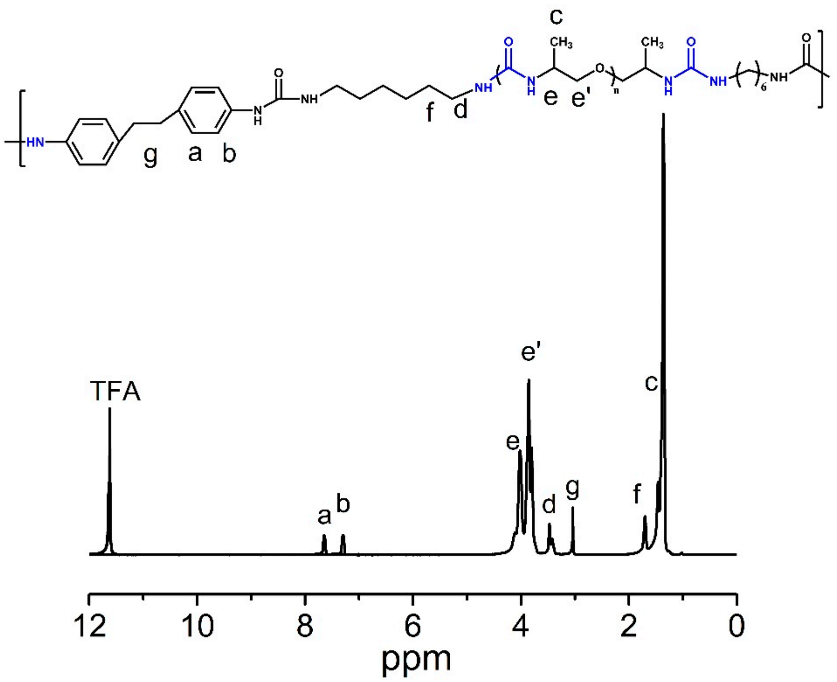

Figure S4. ^{1}H NMR spectrum (in CF3COOD) of PU-H-M.

Table S1. Molecular weights of samples.

Samples	PU-IP-A	PU-IP-M	PU-H-A	PU-H-M*
M_n (g/mol)	12617	12883	15044	--
M_w (g/mol)	18586	19918	18038	--
Mw/Mn	1.47	1.55	1.22	--

***insoluble in THF**

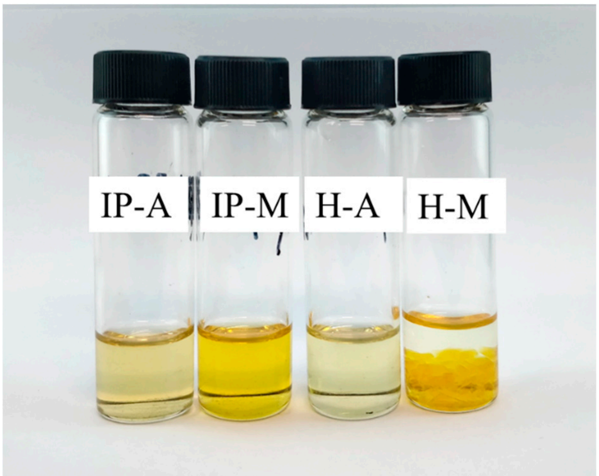

Figure S5. Different solubilities of the four polyureas in DMF after 24 h.

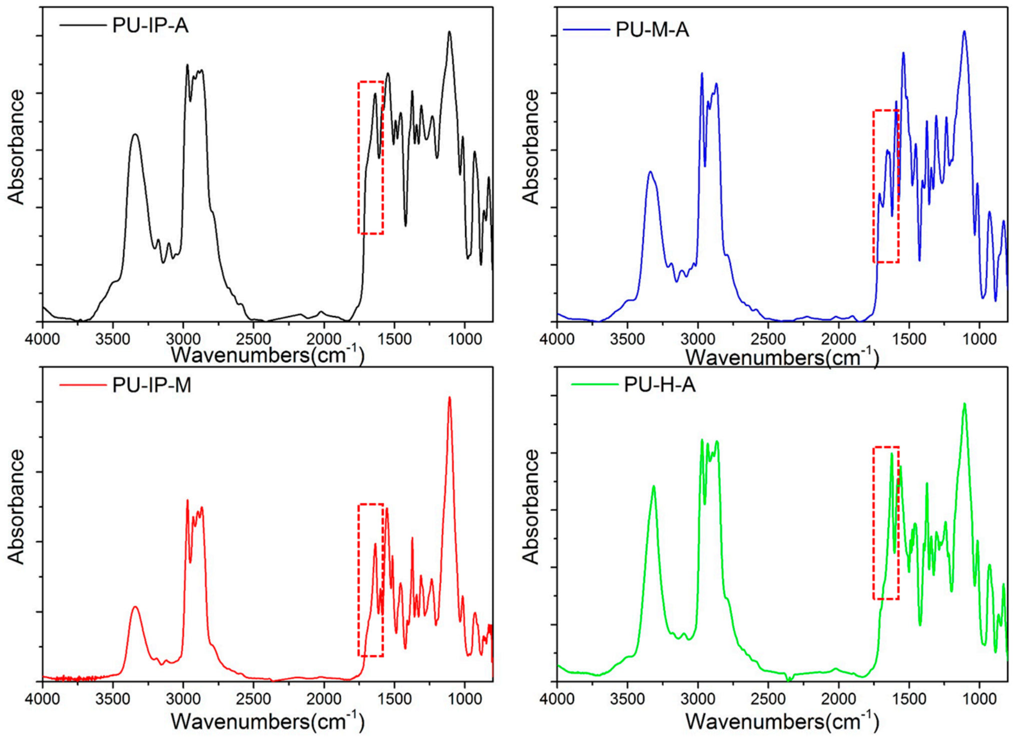

Figure S6. FTIR spectra of PU-IP-A, PU-IP-M, PU-H-M, and PU-H-A.

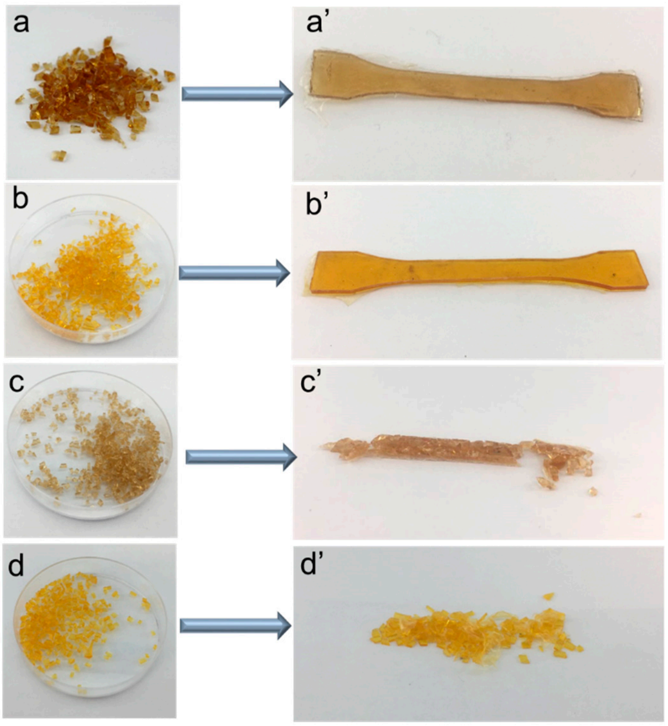

Figure S7. Mechanical reshaping of different samples, (a)~(d) particles that cut from the PU-IP-A, PU-IP-M, PU-H-A, and PU-H-M, respectively, (a')~(d') the obtained specimens after molding by hot pressing at 100 °C for 5 min.

TGA profiles

TGA curves are shown in Figure S8, confirming that the introduction of disulfide bonds would not lead to a decline of the initial decomposition temperatures.

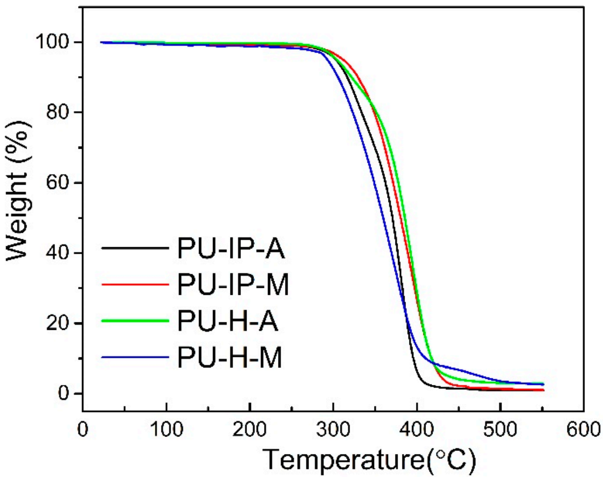

Figure S8. TGA profiles for the four samples.

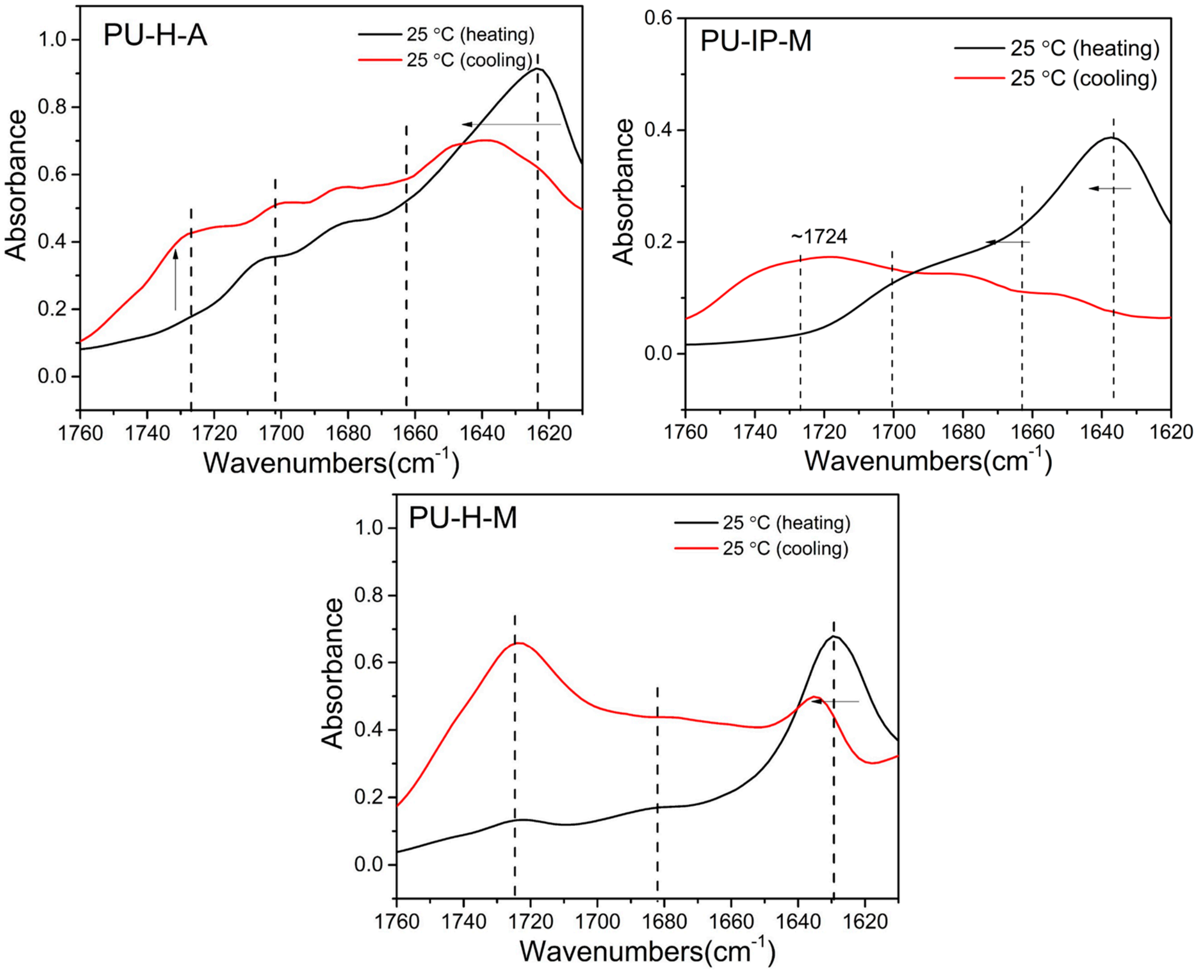

Figure S9. FTIR spectra showing carbonyl groups of PU-IP-M, PU-H-A and PU-H-M at 25 °C before heating and after cooling to 25 °C.

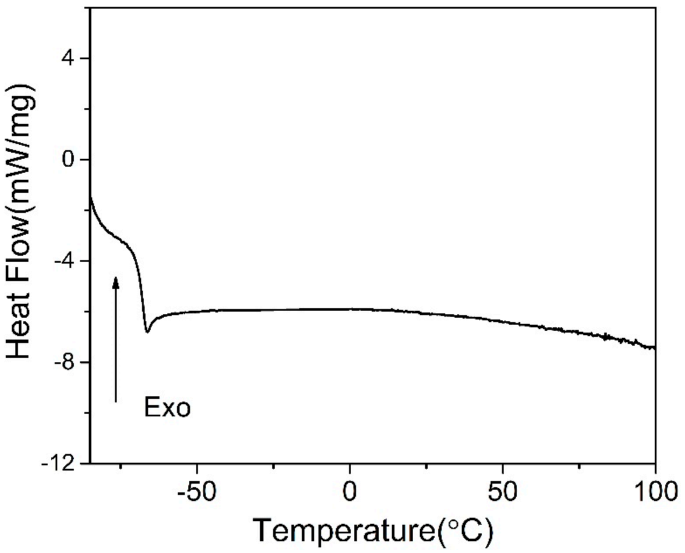

Figure S10. DSC curve of the soft constituent D2000.
